# Fabrication of a Biocompatible Mica/Gold Surface for Tip‐Enhanced Raman Spectroscopy

**DOI:** 10.1002/cphc.201901002

**Published:** 2020-01-08

**Authors:** Xiao You, Clayton B. Casper, Emily E. Lentz, Dorothy A. Erie, Joanna M. Atkin

**Affiliations:** ^1^ Department of Applied Physical Science University of North Carolina at Chapel Hill Caudill Labs, Chapel Hill North Carolina 27514 U.S.A.; ^2^ Department of Chemistry University of North Carolina at Chapel Hill Caudill Labs, Chapel Hill North Carolina 27514 U.S.A.

**Keywords:** biocompatible surface, biological imaging, chemical imaging, DNA, tip-enhanced Raman spectroscopy

## Abstract

Tip‐enhanced Raman spectroscopy (TERS) is a promising technique for structural studies of biological systems and biomolecules, owing to its ability to provide a chemical fingerprint with sub‐diffraction‐limit spatial resolution. This application of TERS has thus far been limited, due to difficulties in generating high field enhancements while maintaining biocompatibility. The high sensitivity achievable through TERS arises from the excitation of a localized surface plasmon resonance in a noble metal atomic force microscope (AFM) tip, which in combination with a metallic surface can produce huge enhancements in the local optical field. However, metals have poor biocompatibility, potentially introducing difficulties in characterizing native structure and conformation in biomolecules, whereas biocompatible surfaces have weak optical field enhancements. Herein, a novel, biocompatible, highly enhancing surface is designed and fabricated based on few‐monolayer mica flakes, mechanically exfoliated on a metal surface. These surfaces allow the formation of coupled plasmon enhancements for TERS imaging, while maintaining the biocompatibility and atomic flatness of the mica surface for high resolution AFM. The capability of these substrates for TERS is confirmed numerically and experimentally. We demonstrate up to five orders of magnitude improvement in TERS signals over conventional mica surfaces, expanding the sensitivity of TERS to a wide range of non‐resonant biomolecules with weak Raman cross‐sections. The increase in sensitivity obtained through this approach also enables the collection of nanoscale spectra with short integration times, improving hyperspectral mapping for these applications. These mica/metal surfaces therefore have the potential to revolutionize spectromicroscopy of complex, heterogeneous biological systems such as DNA and protein complexes.

## Graphical abstract

**Bio‐enhancement**! By combining the biocompatibility of mica surfaces with the field‐enhancement provided by metals, we have designed substrates that increase sensitivity for TERS bio‐imaging by up to five orders of magnitude.

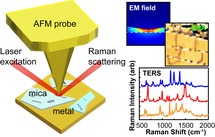

Tip‐enhanced Raman scattering (TERS) combines atomic force microscopy (AFM) with Raman vibrational spectroscopy. AFM is a powerful technique for imaging on the single‐molecule level,^1^ and Raman spectroscopy can distinguish biological constituents and their organization and conformation through their vibrational signatures.^2^, ^3^ TERS therefore has significant potential for resolving open questions in heterogeneous, multicomponent biological systems such as DNA‐protein complexes for transcription and repair.^4^, ^5^, ^6^, ^7^, ^8^, ^9^, ^10^, ^11^, ^12^ However, there remain significant challenges to its widespread implementation and capacity to reliably address biological and clinical questions.

The sensitivity of TERS arises from the excitation of a localized plasmon resonance in a noble metal AFM tip, which can provide an order of magnitude enhancement in the optical field close to the tip. Most TERS studies are additionally performed on noble metal surfaces,^8^, ^12^, ^13^, ^14^ where plasmonic coupling between tip and surface further increases the optical field enhancement. This approach yields single‐molecule and even intra‐molecular sensitivity in small organic molecules.^15^, ^16^, ^17^ Biological macromolecules typically possess low Raman activities, and therefore resolving most biomolecular signatures with TERS also requires strong field enhancement, but metal surfaces are not biocompatible. Metal surfaces are hydrophobic, leading to difficulty in deposition,^18^ and can have a disruptive effect on the native protein or nucleic acid structure.^12^, ^19^ For example, surface‐enhanced Raman spectroscopy (SERS) studies have revealed that the interactions with the plasmonic metal surface promote protein denaturation and DNA double strand breaks.^20^

Muscovite mica, a layered phyllosilicate mineral, is the accepted surface for AFM studies of biomolecules, providing minimal perturbation of the biomolecular structure and atomic flatness.^21^ Its insulating nature, however, reduces the optical field enhancements and spatial resolution for TERS experiments^11^, ^22^ The relatively low sensitivity of TERS with mica surfaces reduces the general applicability of the technique. The use of TERS for understanding complex biological systems therefore calls for the development of an atomically flat biocompatible surface simultaneously capable of high optical field enhancement. Here, we design such TERS surfaces by exfoliating mica onto a noble metal surface, producing a mica/metal/epoxy/base sandwich structure. With this design, the sample is in direct contact with a thin layer of mica and not the destabilizing metal surface, but still benefits from the field enhancement of the metal, as confirmed with electromagnetic (EM) simulations. We demonstrate the minimally‐perturbing nature of the surface using DNA as a test sample, as DNA is one of the most demanding samples with regards to deposition due to its surface properties and small diameter (<1 nm). Within the mica/Au surface we can achieve enhancements in the TERS signal of up to 5 orders of magnitude, while maintaining biocompatibility.

To evaluate the feasibility of our approach, we model the EM field in the gap between a Au AFM tip and various substrates using finite‐element simulations (COMSOL Multiphysics, Wave Optics). In Figure 1, we compare the EM enhancements for a Au tip (modeled as a nanoparticle) with Au surface (Figure 1a), mica/metal surfaces (Figure 1b,c) (1 nm and 5 nm mica on Au, respectively) and bulk mica (Figure 1d). The 2D maps show the spatial distribution of the field enhancement factor, the ratio of the scattered electric field to incident (excitation) field, $E.F.=\frac{\left|E_{scat}\right|}{\left|E_{exc}\right|}$ . As expected, the maximum *E. F*. is found between the tip and surface, and rapidly decays as the tip‐surface separation increases.^23^, ^24^, ^25^

**Figure 1:**
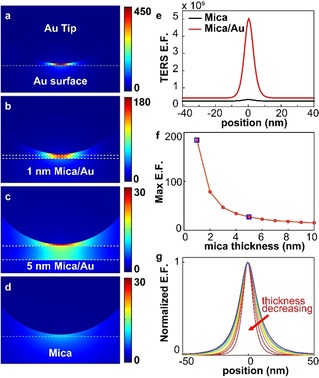
EM field maps for a Au tip (modeled as a nanoparticle) in close proximity to a Au surface (a), 1 nm mica/Au surface (b), 5 nm mica/Au surface (c), and bulk mica (d). Simulations are performed for a tip‐sample distance of 0.5 nm using COMSOL Multiphysics. e) Cross section of TERS enhancement factor (E.F.) on 5 nm mica/Au and mica surfaces. f) Dependence of field E.F on mica thickness; blue squares represent the thickness in (b) and (c). g) Cross section of normalized TERS E.F. on mica of various thickness.

While the noble metal AFM tip provides a strong optical field enhancement at the apex due to the plasmonic response, the properties of the surface are vital for achieving sensitivity.^26^, ^27^, ^28^, ^29^ Similar to gap‐mode plasmons in SERS,^30^ a Au tip in close proximity to a Au surface yields significant improvements in field enhancement over an isolated tip or a tip close to an insulator. Based on our calculations, at a tip‐surface distance of 0.5 nm, the field enhancement for the Au tip/Au surface configuration is 50 times higher than that of Au tip/bulk mica. (Figure 1a,d). As expected, based on SERS substrates,^31^ metal surfaces with an ultrathin dielectric layer such as mica also provide an appreciable field enhancement, which approaches 40 % of the Au/Au system for a 1 nm layer (Figure 1b,c).

Figure 1e shows a line cut of simulated TERS intensity as a function of horizontal position, for a Au nanoparticle above a mica (black) and a 5 nm mica/Au (red) surface at a tip‐surface separation of 0.5 nm. The intensity of the TERS signal (TERS E. F.) scales as (E.F.).^4^, ^23^ The maximum TERS enhancement of 5×10^5^ is over an order of magnitude greater than bulk mica (SI Table 1 in the Supporting Information). The field E.F. decreases exponentially with increasing thickness of mica (Figure 1f). The ideal case of monolayer mica (thickness ∼1 nm) represents the upper limit of enhancement from the system, with an E.F. of approximately 180, corresponding to a TERS enhancement of ∼10^9^ – only an order of magnitude lower than a bare Au surface (SI Table 2). In addition to the high achievable enhancement with thin layers of mica, we observe that the full width at half maximum of the field enhancement (Figure 1g) is under 10 nm and inversely correlated with mica thickness, suggesting that the EM field is highly confined between the tip and surface. The lateral distribution of the optical field determines the spatial resolution of a TERS measurement.^28^ This large field confinement allows for high spatial resolution TERS mapping, potentially surpassing the achievable resolution for electrical AFM techniques.^32^

In addition to reducing the magnitude of the enhancement, a thin dielectric layer leads to shifts in the spectral behavior of gap‐mode plasmons. Figure S1a shows the simulated dependence of the field enhancement on incident wavelength on Au and mica/Au surfaces. Compared to a bare Au surface, adding a thin layer of mica redshifts the surface plasmon resonance peak to around 620 nm, close to the He−Ne laser line at 633 nm, enabling a near resonance condition between tip and surface. The degree of overlap between the excitation wavelength and tip‐surface resonance depends also on the metals used. We have calculated E.F. values (SI Table 3) for different tip and substrate metals at 633 nm. The results show that Cu provides a comparable enhancement for Au, and other metals could also provide an enhancing surface. These results suggest that the mica/metal system offers additional tunability that could make reaching resonant conditions more feasible, given different excitation wavelengths.

Mica's layered crystal structure allows for mechanical exfoliation, similar to that developed in 2D materials including graphene and transition metal dichalcogenides. We therefore adopt fabrication procedures based on exfoliation and template stripping to produce TERS surfaces.^33^ We show a schematic of TERS using the new mica/Au surfaces in Figure 2a, and the fabrication procedure in Figure 2b. Au is sputtered onto a freshly cleaved mica sheet inside a vacuum chamber and then immediately glued to a support (either cover glass or silicon) using low viscosity epoxy. Once the epoxy is fixed, the attached mica sheets are cut into small pieces. A clean surface for TERS can then be obtained by stripping off the top layers of mica with tape before use. Scanning electron microscope (SEM) images (Figure S1) and energy dispersive x‐ray analysis (EDX) (SI Table 4) results confirm that after the initial lift‐off procedure, mica layers of varying thickness remain on the Au surface.

**Figure 2:**
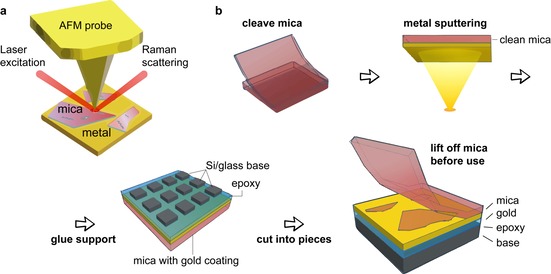
a) Schematic of TERS with mica/Au substrate; b) Illustration of the fabrication procedure of mica/Au substrates.

In general, all surfaces will be quickly covered by a layer of hydrocarbons when exposed to air, which can be detrimental to AFM imaging.^33^ The mica/metal substrates are shelf stable in ambient conditions and can be exfoliated immediately before deposition, providing atomically flat mica surfaces over several hundreds of square micrometers, free of contamination. In TERS experiments, the TERS activity is often affected by the oxidation of reactive metals like silver or the aggregation of noble metal nanostructures like gold nanoparticles.^34^ By cleaving the layered mica crystals just prior to specimen adsorption, we are able to conveniently obtain an active surface that also acts as a protective layer for the metal underneath.

With repeated exfoliation, we can cleave these mica layers further, reaching thicknesses as down to a few layers or even a single layer. Figure 3a shows AFM topographic images of a mica region with a 5 nm thickness (∼5 layers), thin enough to obtain field enhancement from the Au underneath. Micro‐Raman spectra at various positions on a mica/Au substrate (Figure S3) using 633 nm as the excitation wavelength show that the thicker mica flakes (>10 nm) have vibrational modes at 387 cm^−1^, 407 cm^−1^ and 700 cm^−1^ in their Raman spectra. The mica vibrational modes are usually undetectable on layers below 10 nm thick.^33^

**Figure 3:**
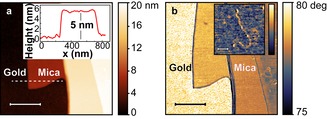
a) AFM topographic image of an edge of a mica flake after tape exfoliation. Inset: height profile along the line marked in (a), showing a thickness of ∼5 nm. b) AFM phase image of mica/Au surface after DNA deposition, showing no trace of DNA on Au, and unconjugated DNA on mica. Inset: High resolution AFM image of an individual double‐strand DNA on mica/Au surface. Scale bars in (a), (b)=500 nm; inset in (b)=100 nm.

Photoluminescence (PL) measurements of mica/Au surfaces (Figure S2b) show a slight redshift of the PL peak in comparison to the Au surface, consistent with the simulated results. TERS enhancement could be optimized by matching the energy of the excitation laser with the coupled tip‐surface plasmon resonance.

The mica/Au surface obtained by the template‐stripping method is shown in the optical image in Figure S4. The large continuous mica area for the template‐stripped mica/Au substrates also alters the hydrophilicity of the surface, with a significantly smaller contact angle than that on a Au surface without mica (Figure S4). As most biological samples are dispersed in aqueous solution, a hydrophilic surface with small contact angle is favorable for sample deposition.

We demonstrate the biocompability of our surface design by depositing DNA on both a mica/Au and a template‐stripped ultraflat Au surface.^35^ For the large area mica/Au surfaces, standard sample preparation techniques for AFM on mica surfaces can be employed.^18^ Unlike proteins, DNA molecules do not adhere to surfaces easily. However, the negatively charged mica surface allows for the immobilization of DNA with the assistance of bridging, divalent cations such as Mg^2+^, Ca^2+^, and Ni^2+^.^36^ Using a low‐salt buffer, we deposited DNA onto our mica/Au surface. Although the ultraflat Au surface exposed during our substrate fabrication has a similar roughness to the mica, no linear DNA strands could be resolved by AFM (Figure S5). Instead, features with ∼10 nm height were observed. This is due to the hydrophobicity of the Au surface, which causes the aqueous sample to not disperse and the biomolecules to coagulate. In contrast, the DNA on the mica/Au section of the surface is well distributed with its linear shape preserved (Figure 3b). Thus, our substrates allow deposition of DNA with little aggregation or change in conformation.

In order to test the achievable field enhancements compared to simulations, TER spectra were collected on a ∼5 nm thick mica/Au surface with a DNA deposited sample. An approach curve was recorded (Figure 4a) as the tip‐sample distance was reduced from 50 nm to contact. The integrated Raman intensity increases dramatically when the tip‐sample distance is under 10 nm (Figure 4b), which is strong evidence of near‐field effects, *i. e*. enhancement due to tip‐surface coupling.^37^ Furthermore, when the tip is in contact with the sample, multiple Raman modes appear in the spectrum that cannot be detected when the tip is retracted (Figure 4c). An experimental contrast factorC of 12.3 was derived by dividing the fitted peak area around 950 cm^−1^ of the tip‐in spectrum by the tip‐out spectrum. The contrast factor represents the increase in signal from a TERS process compared with normal Raman scattering, and our contrast factor is comparable to previous experiments on bare Au surfaces.^12^ Our value for C represents a strong confinement of the TERS signal and demonstrates high spatial and spectral resolution.

**Figure 4:**
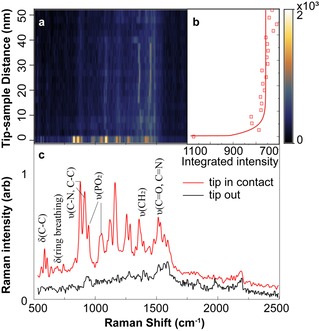
a) Approach curve of Raman intensity recorded as the tip approaches the sample surface, showing the appearance of DNA vibrational modes (800–1300 cm^−1^) when the tip is in close proximity to the sample. b) Integrated intensity versus tip–sample distance. c) TERS of DNA, for tip in contact with surface (red) and tip retracted from the surface (black).

Many factors, such as the tip geometry, preparation, roughness, and the alignment and focus of the incident laser light, affect the degree of enhancement obtained in a TERS measurement, making comparison between measurements and across substrates challenging. Nonetheless, we consistently observed high signal to noise ratios (SNRs) for samples on thin mica in comparison to mica layers greater than 10 nm thick. For example, the SNR for the spectrum in Figure 4c is over 100, calculated by dividing maximum peak height by the RMS noise of the background. In general, with an integration time of 1 second and laser power of ∼0.1 mW we consistently achieved SNRs of 100–200. In contrast, we typically could not detect a TERS signal on DNA on thick mica surfaces, with 25 being the best SNR recorded for a 5 s integration time. The mica/Au surfaces therefore impart an improvement in SNR of at least a factor of 10. This high spectral sensitivity arising from gap‐mode TERS on mica/Au surfaces enables the observation of not only the signature bands corresponding to phosphodiester groups (850–1100 cm^−1^) and C=C, C=N, C=O stretching (1400–1600 cm^−1^), but also the relatively weaker ring‐breathing modes (600–800 cm^−1^) corresponding to nucleotide bases.

We perform hyperspectral TERS mapping to demonstrate the feasibility of scanning with the spatial resolution and sensitivity of mica/Au surfaces. With 1 s accumulation times for each pixel, we achieve a SNR of approximately 100, sufficient to enable imaging of ∼100 nm biomolecular structures within a few minutes. AFM topography (Figure 5a) and phase (Figure 5b) images show a region with mica terraces with thickness from 4–10 nm on Au, with several unconjugated, linear DNA molecules. We collected TER spectra over a smaller region, 250×80 nm^2^, where a strong spectral response was observed on a DNA molecule, as shown in Figure 5c overlaid with the AFM phase image. For an individual DNA strand (measured along the black line in Figure 5c), the spectra demonstrate a high degree of consistency and reproducibility, with intense spectral features that can be assigned to vibrational modes of nucleotide bases and the DNA backbone (Figure 5d). Strong phosphodiester vibrations appear below 1100 cm^−1^. As the breakdown of DNA double strands result in −PO_3_H and −CH_2_/−CH_3_ modes in the 1100–1200 cm^−1^ spectral range,^22^ the presence of the phosphodiester vibrational modes and absence of P‐OH bending and P−O−H stretching modes indicates that the DNA is chemically stable during the measurement.

**Figure 5:**
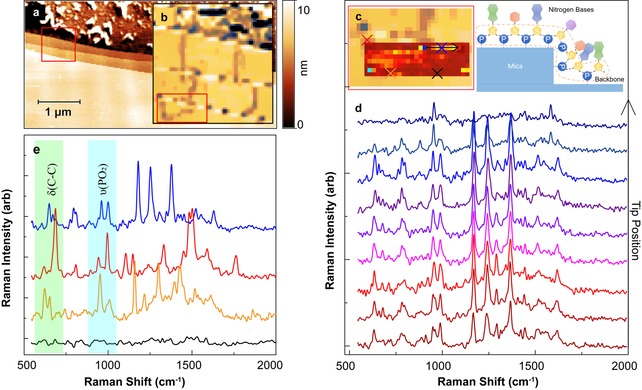
a) AFM topographic image of a mica/Au interface. b) AFM phase image of the red square region in (a), showing multiple DNA strands and mica/Au edges. c) TERS image of a DNA molecule marked in (b) overlaid with a zoomed in phase image of the DNA strand. The TERS image was constructed using principal component analysis to compress the three‐dimensional data. Schematic shows DNA orientation at a mica terrace. d) TER spectra along a DNA strand marked with the black arrow in (c). e) TER spectra of DNA from selected spots in (c).

While spectra within a strand of DNA are remarkably consistent, there are spectral variations between DNA strands (Figure 5e). The spectral features below 850 cm^−1^ (green box) include the fingerprint region of DNA, containing the sensitive ring breathing modes of adenine (A), guanine (G), cytosine (C), and thymine (T), suggesting that this spectral region could be used as a marker to map nucleotide base distribution. The TER spectrum plotted in blue has very similar Raman peak locations and intensities to those observed in a single‐stranded RNA homopolymer of cytosine in a previous study.^11^ With further high‐sensitivity study of DNA/RNA homopolymers, it would be plausible to establish a standard in distinguishing all base pairs in DNA/RNA with TERS.

The relative intensity of Raman peaks in the range of 800–1100 cm^−1^ also changes significantly for the three representative spectra. The Raman modes in this range can be attributed to the DNA backbone, composed of sugar and phosphodiester groups. The phosphate modes are insensitive to polarization, but the deoxyribose ring stretching and C−O stretching are influenced by orientation. The TER spectra from the DNA strand near a mica edge (blue in Figure 5e) has strong spectral features in the 1200–1500 cm^−1^ range which are not observed for the other two DNA strands. This may be due to the DNA backbone adopting a more vertical orientation at the edge, in comparison to the horizontal orientation far from edges, as shown in Figure 5c. The molecular planes of the nucleotide bases are perpendicular to the backbone, and therefore also change orientation at the edges, modifying the vibrational modes excited in the nucleotides. Overall, the high SNR of the spectra enable us to distinguish different spectral regions and their spatial variation with few‐nanometer spatial resolution.

The results presented here demonstrate the functionality and TERS activity of our mica/Au substrates. By combining the biocompatibility of mica surfaces with the field‐enhancement provided by metals, we have designed substrates that increase sensitivity for TERS bio‐imaging by up to five orders of magnitude. The substrates have atomically flat surfaces that are hydrophilic, facilitating the deposition of aqueous samples following standard protocols developed for AFM studies. These substrates require little to no surface preparation and a clean surface can be obtained by exfoliating the mica layer with adhesive tape prior to sample deposition. Numerical simulations of mica/Au substrates illustrate the formation of coupled plasmon enhancements from the probe and the mica/metal substrates. This was experimentally confirmed by collecting TER spectra of DNA on one of these new surfaces, which displayed good deposition, high sensitivity, and high spatial resolution. Due to protection from the top layer, these substrates have an extended shelf life compared to reactive metals. This is extremely valuable for Ag, which can exhibit stronger plasmonic activity than Au but tends to oxidize in just a few hours when exposed in air.

These surfaces enable the identification and structural characterization of heterogeneous biomolecular systems with sensitivity improved by an order of magnitude. TERS spectra can be reproducibly obtained with high contrast factors and short integration times. Combined high resolution topographic and phase information with TERS on these surfaces demonstrates a critical first step to resolve long‐standing problems in protein‐nucleic acid interactions, protein aggregation and proto‐fibril formation. These mica/metal substrates can also be utilized for other near‐field microscopy techniques, such as infrared scattering near‐field optical microscopy (s‐SNOM), where a field enhancement is also beneficial. The presented Au/mica preparation approach could also benefit surface plasmon resonance spectroscopy, where biomolecule binding properties at noble metal surfaces are frequently investigated under non‐bio‐native conditions. The development of biocompatible surfaces for nanospectroscopy, with sensitivity up to five orders of magnitude greater than previously demonstrated, therefore has the potential to yield revolutionary insights into the emergence of higher order structure and how nanoscale organization relates to function, pushing the frontier of modern bio‐imaging.

## Supporting information

As a service to our authors and readers, this journal provides supporting information supplied by the authors. Such materials are peer reviewed and may be re‐organized for online delivery, but are not copy‐edited or typeset. Technical support issues arising from supporting information (other than missing files) should be addressed to the authors.

Supplementary
