# Supplementary material for: Fabrication of a Biocompatible Mica/Gold Surface for Tip‐Enhanced Raman Spectroscopy
Source: Chemphyschem. 2020 Jan 8;21(3):188–93. doi: 10.1002/cphc.201901002 (PMC7027439; doi:10.1002/cphc.201901002)
Supplement: Supplementary file 1 — Supplementary [file CPHC-21-188-s001.pdf]

CHEMPHYSCHEM

Supporting Information

© Copyright Wiley-VCH Verlag GmbH & Co. KGaA, 69451 Weinheim, 2020

Fabrication of a Biocompatible Mica/Gold Surface for Tip-Enhanced Raman Spectroscopy

Xiao You, Clayton B. Casper, Emily E. Lentz, Dorothy A. Erie, and Joanna M. Atkin*© 2020 The Authors. Published by Wiley-VCH Verlag GmbH & Co. KGaA. This is an open access article under the terms of the Creative Commons Attribution License, which permits use, distribution and reproduction in any medium, provided the original work is properly cited.

Supporting Information

Experimental Section

Fabrication of Template-stripped mica/Au:

Muscovite mica sheets (Electron Microscopy Sciences, Hatfield, PA; Spruce Pine Mica Company, Spruce Pine, NC) were cleaved using scotch tape and loaded into a Kurt Lesker PVD 75 chamber. 50 nm gold was sputtered under $5x10^{-5}$ Pa at a rate of 2 Å/s. The base of the substrate (cover glass or silicon) was prepared by cutting the material into 1 cm^2 pieces followed by rinsing and UV-ozone cleaning. The bases were then glued to the gold coated mica sheets immediately after sputtering using low viscosity epoxy (H70S, Epoxy Technology Inc, Billerica, MA). The product was heated at 120 °C for 2 hours and then cut into small pieces. The top most layer of mica was lifted off immediately before use.

DNA preparation and purification:

Plasmid (pUC19-VSR) was purified from *E. coli* using a plasmid mini kit (QIAGEN, Hilden, Germany). The plasmid was digested with the restriction enzyme XmnI (New England Biolabs, Ipswich, MA), resulting in linear DNA with a length of 2708 base pairs. It was then purified with a PCR purification kit (QIAGEN, Hilden, Germany).

DNA deposition:

For imaging, purified linear DNA was diluted to a concentration of 1-5 ng/µL in a low salt imaging buffer of 25 mM HEPES, pH 7.5, 50 mM $NaCH_3COO$, and 10 mM $Mg(CH_3COO)_2$. Linear DNA was deposited onto a freshly cleaved mica/Au surface at room temperature, washed with pure water (Sigma Aldrich, St. Louis, MO), blotted dry, and further dried under a gentle flow of nitrogen.

TERS probes:

TERS active probes were prepared by coating 80 nm of gold onto ultrasharp noncontact silicon tips (NSC12, MikroMasch, Watsonville, CA). The coating was sputtered under $5x10^{-5}$ Pa at a rate of 1.5 Å/s.

AFM imaging:

Figure 3 in the main text was obtained by imaging a DNA deposited sample in air with an Asylum MFP-3D AFM (Asylum Research) in AC mode. Scout 350 AC mode silicon probes (Nu Nano Ltd, Bristol, UK) with spring constants of ~42 Nm^{-1} and resonant frequencies of ~350 kHZ were used. The AFM height and phase images in Fig.5 were obtained with AIST-NT SmartSPM scanning probe microscope (Horiba). A gold coated TERS tip was used. All images were collected at a scan rate of 1 Hz and a resolution of 256x256 pixels. All AFM images were processed with Gwyddion software. Processing methods include aligning rows, leveling data and shifting minimum data value to zero. Height profiles are also extracted using Gwyddion then plotted with OriginLab software.

Tip-enhanced Raman spectroscopy:

Our TERS experiments were performed with the AFM-Raman system from Horiba, comprising an AIST-NT SmartSPM scanning probe microscope, equipped with a side-illumination Raman LabRAM HR Evolution. The sample is illuminated from the side, and the Raman spectrum is collected through the same objective lens (50x magnification, NA 0.5, Olympus). A HeNe laser with the wavelength of 633 nm was used for excitation. The laser power at the sample was less than 0.4 mW to avoid damage to the sample and tip. Each pixel on the TERS map is from 1s accumulation. The TERS data is processed with Matlab. Processing methods include normalization, background subtraction and spike removal. MATLAB HYPER-Tools Toolbox is used to extract information from TERS hyperspectral image. The color map in Figure 5c in the main text is obtained through a principal component analysis (PCA) algorithm.

COMSOL simulations:

The tip was approximated as a Au sphere with $r = 25$ nm above a thin layer of muscovite mica covering a metal substrate. The refractive indices at $\lambda = 632.8$ nm used for Au,[1] and mica[2] are $\tilde{n}_{Au} = 0.17 + i3.47$, and $\tilde{n}_{mica} = 1.60 + i0.00$ respectively. These refractive indices from refs. 1 and 2 were also used for the wavelength sweep. The background electric field E_{ref} was calculated in the absence of the Au nanoparticle using a 3-D, 2-port model with Floquet periodic conditions on the side boundaries. The top port simulated a TM plane wave with incident power $P_i = 1.1$ µW and $\theta_i = 30°$. The scattered electric field E_{scat} from the Au nanoparticle was simulated as a perturbation to E_{ref}, with enhancement factors defined as $E.F. = \frac{|E_{scat}|}{|E_{ref}|}$. The accuracy and computation time of the simulation is directly related to the minimum element size, which was set to $l_{min} = 1$ nm and 7.5 nm for the $d = 0.5$ nm and $d = 5$ nm case respectively.

Other measurements:

The SEM and EDX measurements were taken on Hitachi S-4700 Cold Cathode Field Emission Scanning Electron Microscope. The Raman and photoluminescence measurements were conducted using the Horiba LabRam Raman spectrometer, using a 632.8 nm laser. All the Raman and PL spectra were collected through an 100x objective (0.9 NA, Olympus).

**Distance**	d = 5 nm				d = 0.5 nm			
**Surface**	Au	Mica	Mica/Au	Mica/Ag	Au	Mica	Mica/Au	Mica/Ag
**Field E.F**	10	5	7	8	459	10	27	25
**TERS E.F**	1×10^4	625	2401	4096	4.4×10^{10}	1×10^4	5.3×10^5	3.9×10^5

SI Table 1. Enhancement factors calculated with COMSOL Multiphysics at 30 degrees incidence, wavelength of 632.8 nm and a mica layer thickness of 5 nm. The field E.F was always at maximum directly underneath nanoparticle.

**Mica Thickness (nm)**	1	2	3	4	5	6	10	bulk
**Field E.F**	184	79	47	34	27	23	15	10

**TERS E.F.**	1.1×10^9	3.8×10^7	4.8×10^6	1.4×10^6	5.6×10^5	2.7×10^5	5.5×10^4	1×10^4

SI Table 2. Enhancement factors calculated with COMSOL Multiphysics with varying mica layer thicknesses. Tip-sample distance is 0.5 nm; wavelength is 632.8 nm.

	**Ag**[3]	**Au**[1]	**Cu**[3]	**Pt**[4]	**Pd**[4]	**Al**[5]
**Ag**	19	21	21	18	18	18
**Au**	25	27	27	23	23	22
**Cu**	25	28	28	23	24	22
**Pt**	15	16	16	14	15	14
**Pd**	16	17	18	15	16	15
**Al**	14	15	15	13	14	13

SI Table 3. Permittivity dependence of the field enhancement. Enhancement factors of different metal pairs were calculated with COMSOL Multiphysics at 30 degree incidence, a wavelength of 632.8 nm, and with the metal nanoparticle 0.5 nm above a 5 nm mica/metal surface. Results indicate that the same structure can be extended to metals other than gold.

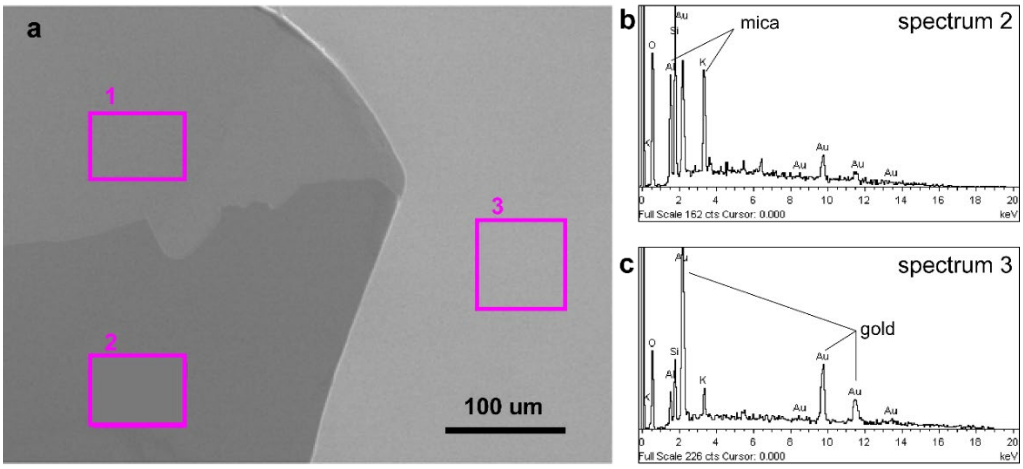

SI Figure 1. a) SEM and b), c) XPS of the mica/Au surface. After the initial liftoff procedure, mica layers of varying thickness remain on the Au surface.

Spectrum 1	Weight%	Atomic%
O K	32.10	74.45
Al K	2.79	3.84
Si K	5.21	6.88
K K	4.65	4.42
Au M	55.24	10.41
Totals	100.00	

Spectrum 2	Weight%	Atomic%
O K	40.61	71.45
Al K	6.62	6.91
Si K	9.38	9.40
K K	10.47	7.54
Au M	32.93	4.71
Totals	100.00	

SI Table 4. XPS of two mica/Au surfaces. The change in elemental composition indicates a varying thickness of the mica layer.

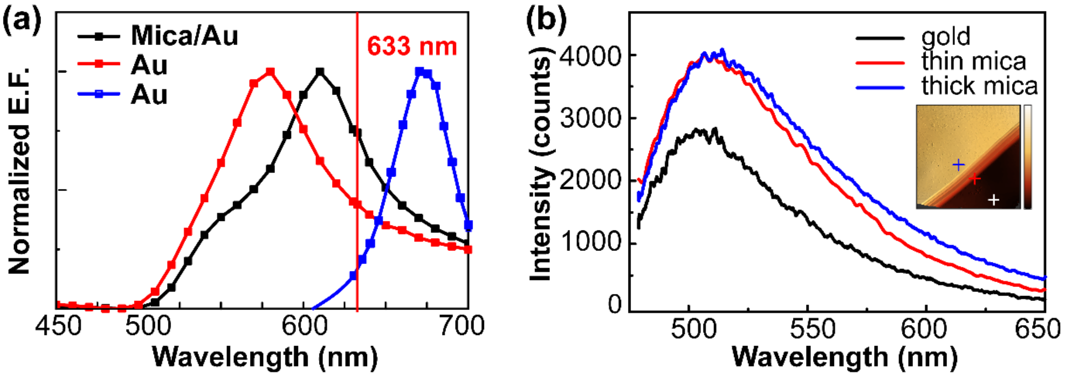

SI Figure 2. (a) Dependence of E.F on excitation laser wavelength. The excitation laser used in TERS measurements is marked as the red line at 633 nm wavelength. Red squares = Au nanoparticle 0.5 nm away from a Au substrate; black squares = Au nanoparticle 0.5 nm away from 1 nm mica/Au; blue squares = Au nanoparticle 1.5 nm away from Au substrate (b) Photoluminescence (PL) measurements of mica/Au surfaces show a slight redshift of the PL peak in comparison to the Au surface. An AFM image of the mica/Au surface is inserted to indicate the sample spots. PL was taken using 473 nm laser as incident light.

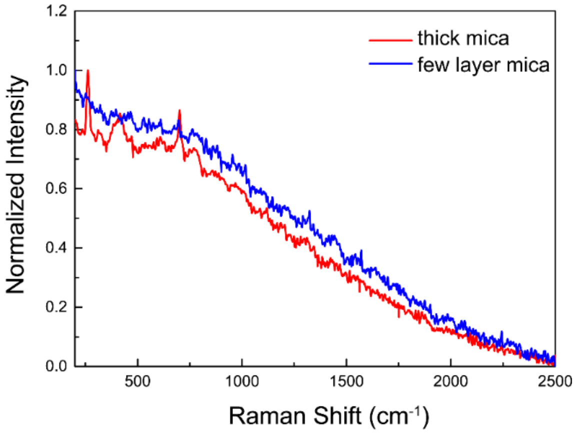

SI Figure 3. Raman spectra taken at various positions on a mica/Au substrate using a 633 nm laser. Raman measurements confirmed that bulk mica flakes (>10 nm) have Raman peaks at 387, 407 and 700 cm^{-1}. At mica thicknesses below 10 nm, we cannot detect these Raman peaks. We also do not observe mica vibrational modes in the TERS spectra, and therefore they do not interfere with the Raman signature of deposited samples.

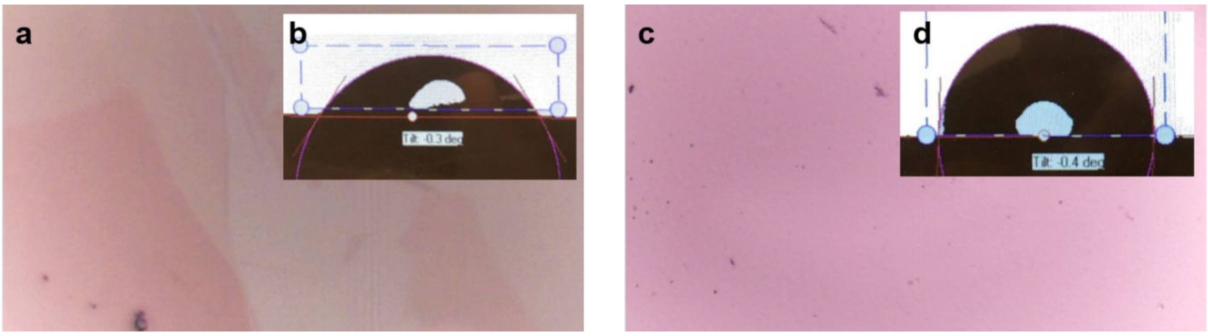

SI Figure 4. a) Optical image and b) contact angle tests of mica/Au surface; c) optical image and d) contact angle tests of ultraflat Au surface.

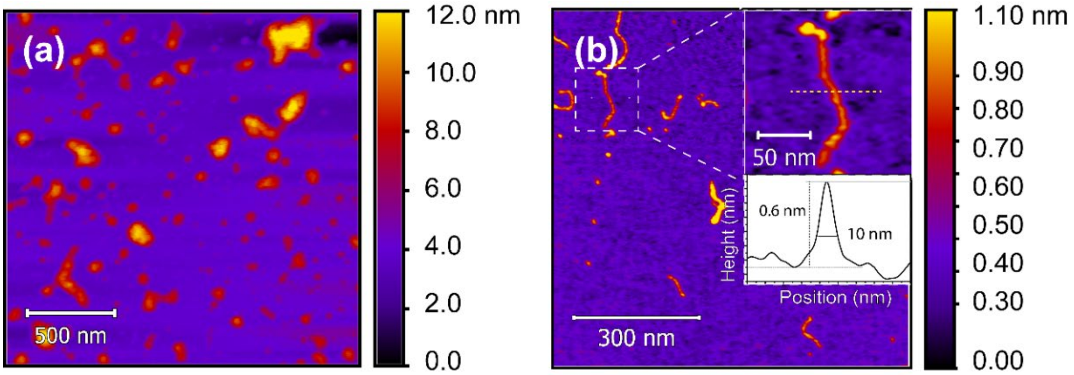

SI Figure 5. AFM image of an (a) ultra-flat Au and (b) plain mica surface after DNA deposition.

References

[1] R. L. Olmon, B. Slovick, T. W. Johnson, D. Shelton, S. H. Oh, G. D. Boreman, M. B. Raschke, *Phys. Rev. B*. **2012**, *86*, 235147.

[2] R. Nitsche, T. Fritz, *Appl. Opt*. **2004**, *43*, 3263-3270.

[3] P. B. Johnson, R. W. Christy, *Phys. Rev. B*. **1972**, *6*, 4370-4379.

[4] W. S. M. Werner, K. Glantschnig, C. Ambrosch-Draxl, *J. Phys. Chem. Ref. Data*. **2009**, *38*, 1013-1092.

[5] A. G. Mathewson, H. P. Myers, *Phys. Scr*. **1971**, *4*, 291.
